# Does the Clinical Context Improve the Reliability of Rheumatologists Grading Digital Ulcers in Systemic Sclerosis?

**DOI:** 10.1002/acr.22833

**Published:** 2016-07-28

**Authors:** M. Hughes, C. Roberts, A. Tracey, G. Dinsdale, A. Murray, A. L. Herrick

**Affiliations:** ^1^ Centre for Musculoskeletal Research, University of Manchester, and Manchester Academic Health Science CentreManchesterUK; ^2^ Centre for Biostatistics, Institute of Population Health, School of Medicine, University of ManchesterManchesterUK; ^3^ Centre for Musculoskeletal Research, University of Manchester, Manchester Academic Health Science Centre, and NIHR Manchester Musculoskeletal Biomedical Research Unit, and Central Manchester NHS Foundation TrustManchesterUK

## Abstract

**Objective:**

Digital ulcers (DUs) are often a primary end point in systemic sclerosis (SSc; scleroderma) clinical trials, although the reliability of rheumatologists grading DUs is poor to moderate at best. DU assessment in recent trials has been based upon visual inspection alone, which potentially misses “real‐world” clinical contextual information. Our aim was to investigate whether this clinical information improves the reliability of rheumatologists grading DUs. A secondary aim was to assess agreement between patients and rheumatologists.

**Methods:**

Eighty images of a range of digital lesions were collected from patients with SSc with the clinical context: pain (severity and temporal relationship), lesion duration, and discharge (patient reported and clinician observed). Raters received all images either with or without the clinical context, and graded these images (using a custom‐built interface) on an ordinal scale of severity: no ulcer, inactive ulcer, or active ulcer. Patients also graded their lesion(s) on the same scale.

**Results:**

Fifty‐one rheumatologists from 15 countries completed the study (26 without and 25 with context): 4,590 (including 510 repeated) image gradings were obtained. Context did not significantly increase (without and with context) either intra‐ (0.64, 0.71) or interrater (0.32, 0.36) reliability. Pain (visual analog scale and temporal relationship) and discharge (patient reported and clinician observed) were associated with increased lesion severity, and duration with reduced severity. Agreement between individual patients and rheumatologists was poor without and with context (0.19, 0.28).

**Conclusion:**

The overall intra‐ and interrater reliability of DU grading did not significantly improve with the clinical context. Agreement between patients and rheumatologists was poor.

## INTRODUCTION

Digital ulcers (DUs) are common in patients with systemic sclerosis (SSc; scleroderma) and are responsible for much of the pain and disability associated with the disease. Half of patients with SSc may report a history of DUs (often early in the course of the disease) 1. These DUs are associated with high levels of hand (and global) disability, impacting negatively on the activities of daily living (including occupation) and health‐related quality of life 2. Patients with DUs have a worse clinical outcome than those without 3, including an association with internal organ involvement in very early SSc 4. Despite effective drug therapies (e.g., endothelial receptor antagonists and phosphodiesterase 5 inhibitors) 5, 6, 7, patients may still develop new DUs on treatment.

Box 1 **Significance & Innovations**

- The overall intra‐ and interrater reliability of digital ulcer grading did not significantly improve with added clinical context.
- There was a trend that some clinicians may use the clinical context to help classify lesions as “no ulcer.”
- Pain (visual analog scale and temporal relationship) and discharge (patient reported and clinician observed) were associated with increased lesion severity, and lesion duration with reduced severity.
- Future research is warranted to improve the reliability of rheumatologists grading digital ulcers as an end point in systemic sclerosis–related clinical trials.

DUs are also important for 2 other reasons: first, they are often a primary outcome measure in SSc‐related clinical trials, and second, “fingertip lesions” (including DUs) are now included in the 2013 American College of Rheumatology/European League Against Rheumatism classification criteria for SSc 8. Of concern, the reliability of rheumatologists with an interest in SSc (i.e., those who are likely to assess patients with SSc in clinical trials) grading DUs has been reported to be poor to moderate at best 9, 10. The assessment of DUs, including in several recent multicenter, placebo‐controlled trials, has generally been based upon visual inspection of the lesion alone 5, 6, 7, 11. A potentially key issue is that this misses important clinical contextual information (e.g., whether the lesion has recently developed associated pain, and whether there has been discharge) that many clinicians use in their routine practice when assessing DUs. If the inclusion of the clinical contextual information were to improve the reliability of DU grading, then its incorporation should be strongly considered in the design of future SSc‐related clinical trials.

Against this background, our primary aim was to investigate the intra‐ and interrater reliability of the grading of photographs of digital lesions (chosen to include mainly lesions likely to be classed as DUs) by rheumatologists with an interest in SSc, both without and with the accompanying contextual clinical information. A secondary aim was to examine the agreement between individual patient and rheumatologist opinion.

## PATIENTS AND METHODS

#### Study design and participants

**Figure 1:**
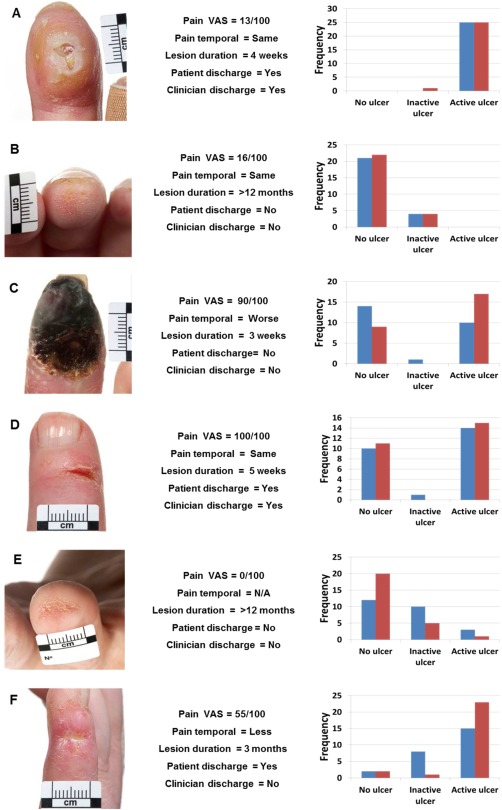
Grading of the images of digital lesions by rheumatologists. Left column shows images of the digital lesions, center column shows clinical contextual information, and right column shows the results of the grading (without clinical context [blue] and with [red]). **A** and **B**, Images with high agreement irrespective of context; **C** and **D**, low agreement (with little improvement with context); and **E** and **F**, substantial change in grading with the context. **A**, An example of an ulcer overlying an area of calcinosis.

Eighty clinical images of a range of digital lesions (location of lesion [number]: fingertip = 34, extensor = 32, and other = 14) were prospectively collected from 36 patients with SSc‐spectrum disorders (the majority of whom had SSc) either at their routine clinic attendance or during an episode of hospitalization. Digital lesions were selected by 2 individuals (MH and ALH) to represent a spectrum of lesions representative of those encountered in patients with SSc. Since the definition of gangrene as ulceration is controversial, one example of gangrene was included 9. A trained medical photographer took all the images, placing a small graded scale (length of 1 cm) in close proximity to the digital lesion to give raters an indication of the size of the lesion. For each digital lesion the following clinical contextual information was collected (exemplified in Figure 1): the pain associated with the lesion on a visual analog scale (100 being most severe) and the temporal relationship (whether the pain was less, the same or worse than a week previously), the duration of the lesion (patient reported), and the presence of discharge (both patient reported and clinician observed). Patient and digital lesion characteristics are presented in Supplementary Table 1 (available on the *Arthritis Care & Research* web site at http://onlinelibrary.wiley.com/doi/10.1002/acr.22833/abstract). The study was approved by the National Research Ethics Committee East of England, Hatfield, and all patients provided signed informed consent.

To facilitate the study, a custom‐built, secure web‐based interface was constructed to display and to record the grading of the images. Individuals (raters) with an interest in SSc (i.e., representative of those clinicians who would be scoring DUs in clinical trials) were invited through SSc‐based organizations (the UK Scleroderma Study Group and the Scleroderma Clinical Trials Consortium) to participate in the study. Raters were randomized to receive all the images either without or with the clinical contextual information. Each rater graded 90 images, 80 unique images (in a random order) and then 10 repeated images (from the first 50 images), to allow an assessment of intrarater reliability. Raters graded the images on a 3‐point ordinal scale of severity: either no ulcer (0) or ulcer, and if ulcer then either inactive 1 or active 2. No exemplar images or definitions (of the grading system) were provided at any point during the study. Raters were not able to return to previously graded images once they had selected their chosen answer for an individual image.

#### Patient grading of digital lesions

At the same time the clinical photograph was obtained, patients were also asked to grade their own digital lesion(s) on the same ordinal scale of severity that was subsequently used by rheumatologists to grade the photographic images. Patients were not given definitions of the different categories of lesion, but graded lesions based on their personal experience.

#### Statistical analysis

Reliability of categorical scales can be assessed using kappa coefficients that calculate the measure of agreement between raters. The categories of the rating scale being used were ordered in terms of severity as no ulcer, inactive ulcer, and active ulcer. For ordered scales, a weighted kappa coefficient can be used, which is also an intraclass correlation coefficient. Overall intrarater reliability was assessed using a weighted kappa coefficient with quadratic weights. Similar to our previous study 9, the reliability of ratings between pairs of categories was assessed using the interclass kappa coefficients 12. We assessed the reliability by combining adjacent categories on the ordinal scale: no ulcer versus ulcer (active and inactive combined), and for no ulcer and inactive ulcer versus active ulcer 9. Although somewhat arbitrary, it has been suggested that the kappa statistic can be interpreted as less than by chance alone (<0.0), poor (0.01–0.20), fair (0.21–0.40), moderate (0.41–0.60), substantial (0.61–0.80), almost perfect (0.81–0.99), and perfect (1.0) agreement between raters 13, so that a change in the kappa statistic of ±0.1 could therefore be considered a meaningful change in reliability. Confidence intervals for the kappa coefficient (means) were generated by the nonparametric bootstrap method, with random resampling (n = 1,000) by rater. The association between the clinical context components was investigated using ordinal logistic regression, and agreement between the individual patient and rheumatologists was explored using a weighted kappa coefficient with quadratic weights. All statistical analyses on the data were performed using STATA, version 13.

## RESULTS

Fifty‐one clinicians (raters) completed the study: 26 without and 25 with the clinical context. The raters came from 15 countries, the majority from the US (33%), UK (22%), Canada (10%), or Italy (8%). A total of 4,590 (4,080 unique and 510 repeated) image gradings were obtained. The mean ± SD score, both without and with the clinical context for the first (unique) images, was 1.08 ± 0.85 and 1.01 ± 0.87 (*P* = 0.32), and for the second (repeat) image was 1.13 ± 0.82 and 1.05 ± 0.90 (*P* = 0.28), respectively. Figure 1 depicts a range of digital lesions demonstrating examples of where there was almost perfect agreement between raters (without or with the clinical context), significant disagreement (without or with the clinical context), and where the grading significantly changed with the context.

#### Intrarater reliability

Intrarater reliability is summarized in Table 1. The overall intrarater reliability (on an ordinal scale of severity) was high with no significant difference observed between those graders without and with the clinical context. The overall weighted kappa coefficient was 0.64 (95% CI 0.53, 0.75) and 0.71 (95% CI 0.64, 0.78) for without and with context respectively. The analyses comparing pairs and when combining adjacent categories is presented in Table 1.

**Table 1:** The intra‐and interrater reliability of the grading of digital lesions, without and with the clinical context^a^

	**Intrarater reliability (95% CI)**		**Interrater reliability (95% CI)**	
	Without context	**With context**	**Without context**	**With context**
Overall	0.64 (0.53, 0.75)	0.71 (0.64, 0.78)	0.32 (0.25,0.39)	0.36 (0.28, 0.44)
Pairwise				
No ulcers vs. inactive ulcers	0.36 (0.08, 0.60)	0.67 (0.50, 0.84)	−0.07 (−0.17, 0.02)	−0.03 (−0.12, 0.05)
No ulcers vs. active ulcers	0.95 (0.90, 1.00)	0.90 (0.84, 0.95)	0.42 (0.34, 0.51)	0.44 (0.35, 0.53)
Inactive vs. active ulcers	0.53 (0.38, 0.67)	0.41 (0.22, 0.60)	0.22 (0.14, 0.30)	0.21 (0.12, 0.30)
Dichotomized				
No ulcers vs. inactive/active ulcers	0.71 (0.60, 0.81)	0.82 (0.75, 0.89)	0.22 (0.16, 0.27)	0.26 (0.21, 0.31)
No ulcers/inactive ulcers vs. active ulcers	0.74 (0.66, 0.82)	0.72 (0.65, 0.79)	0.32 (0.25, 0.39)	0.35 (0.27, 0.43)

a 95% CI = 95% confidence interval.

#### Interrater reliability

Interrater reliability is summarized in Table 1. The overall inter‐rater agreement as measured by a weighted kappa coefficient was poor with no major difference without (κ = 0.32, 95% CI 0.25, 0.39) or with (κ = 0.36, 95% CI 0.28, 0.44) the clinical context. The analyses comparing pairs and when combining adjacent categories is presented in Table 1.

#### Impact of the clinical contextual information

Table 2 presents the results of the ordinal logistic regression (combined/pooled odds ratios) between the individual clinical context components and the overall grading of the digital lesions by raters (including those who did and did not receive the clinical context).

**Table 2:** Ordinal logistic regression between the clinical context and the image grading^a^

Univariate	Without context	With context	Interaction	*P*
Pain VAS†	1.05 (1.03, 1.07)	1.23 (1.18, 1.28)	1.17 (1.12, 1.22)	< 0.001
Pain temporal	1.02 (0.97, 1.06)	1.53 (1.41, 1.67)	1.51 (1.37, 1.66)	< 0.001
Duration of lesion	0.82 (0.79, 0.86)	0.76 (0.71, 0.80)	0.92 (0.86, 0.98)	0.015
Discharge: patient reported	5.37 (4.02, 7.17)	8.64 (6.02, 12.38)	1.61 (1.02, 2.55)	0.042
Discharge: clinician reported	3.00 (2.13, 4.24)	4.21 (2.86, 6.20)	1.40 (0.84, 2.35)	0.200

a Combined (pooled) odds ratios are presented with 95% confidence intervals.

Pain visual analog scale (VAS) given for a 10% increase on a 0–100 scale.

#### Subgroup analysis of digital lesion anatomical location

The clinical context was associated with an increase in the intrarater reliability (without and with, respectively) for fingertip (κ = 0.58 and 0.73) and for other lesion (κ = 0.67 and 0.75) but not of extensor lesions (κ = 0.68 and 0.66). There was, however, no notable change in interrater reliability for fingertip (κ = 0.37 and 0.42), extensor (κ = 0.26 and 0.30), or other (κ = 0.31 and 0.39) digital lesions with the addition of the clinical context.

#### Agreement between the individual patients and rheumatologists

Individual patients and rheumatologists infrequently graded the digital lesions as the same category (on an ordinal scale of severity), without or with the clinical context (42% versus 48%, respectively). There was evidence of a marked disparity between patients and rheumatologists with little (clinically relevant) improvement with the addition of the clinical context (weighted kappa value of 0.19 versus 0.28), which may reflect the uncertainty of the raters, rather than differences of opinion.

## DISCUSSION

The key finding of our study is that adding clinical context did not significantly change the overall intra‐ or interrater reliability of the grading of digital lesions on an ordinal scale of severity by rheumatologists with an interest in SSc. As might be expected and in keeping with our previous study 10, intrarater reliability was significantly higher (both without and with context [0.64 and 0.71, respectively]) than interrater reliability (0.32 and 0.36, respectively). The poor interrater reliability is of concern because this suggests that individuals with an interest in SSc are likely to disagree, even on a 3‐point ordinal scale of severity.

Several patterns emerged from the grading of the images as depicted in Figure 1. First, there were a number of images that the raters were in complete (or almost complete) agreement about the assigned category, particularly at the extremes of the scale (no ulcer or active ulcer), independent of the clinical context (exemplified by Figures 1A and B). Second, there was a group of images in which raters were divided in opinion between the ends of the scale (i.e., no ulcer and active ulcer), with no significant improvement in agreement with the context (exemplified by Figures 1C and D). Third, there were images (exemplified by Figures 1E and F) where the overall lesion grading was shifted (either lower or higher) with the context.

Intrarater reliability was much greater with the addition of clinical context for no ulcers versus inactive ulcers (0.36 versus 0.67) and no ulcers versus inactive ulcers/active ulcers (0.71 versus 0.82), which suggests that some clinicians might use the clinical context in particular to distinguish non‐ulcer lesions from ulcers. This effect is lost when assessing interrater reliability due to the significant disagreement between raters, including a negative kappa statistic for the interrater reliability of no ulcers versus inactive ulcers (which suggests that agreement was less than chance irrespective of context). For inactive ulcers versus active ulcers there was a decrease (0.53 versus 0.41) in intrarater reliability with the addition of context, which suggests that the clinical context might also introduce confusion between adjacent (similar) categories.

As might be expected, pain (visual analog scale and temporal relationship) and discharge (patient reported and clinician observed) were associated with increased severity, and lesion duration with reduced severity. Rater scoring was associated with clinical context (including in those graders who did not receive context), which in part suggests that the clinical context is either visible or associated with other visible features. It is likely that because DU assessment is complex and rheumatologists obtain a wealth of clinical cues from visual assessment of the lesion, that these are interrelated, and that the clinical context may help to inform their classification.

There was a significant increase in intrarater reliability for fingertip lesions with the clinical context (0.58 versus 0.73). Only those DUs that occur at the fingertips are believed to be ischemic 14. Several recent studies have only included DUs that occur distal to the proximal interphalangeal joints (including those at the fingertip) 5, 6, 7; therefore, raters (including through participation in SSc clinical trials) might be more familiar in assessing fingertip lesions.

Agreement between the individual patients and rheumatologists was poor, with only a small increase between those rheumatologists who did not (0.19) or did receive (0.28) the clinical context. This is important for 2 reasons; first, does the patient DU construct potentially hold components that might improve clinician DU assessment? Second, there are implications for clinical practice; for example, does the patient seek medical attention appropriately for the development of DUs and/or treatment escalation?

A North American working group recently developed by consensus a classification of DUs in SSc 10. Although there are important differences between the 2 studies, including the inclusion of healed and indeterminate DUs in the study by Baron et al, the reliability of DU assessment in live patients was not significantly higher compared with our results.

Our study has limitations. This was a web‐based study; therefore, although this approach allowed large numbers of raters to participate, it could be argued that this misses real‐world clinical cues used by clinicians when assessing DUs in routine practice. We used a simple, pragmatic scoring system that did not include either healed or indeterminate categories that have been included in recent multicenter studies 5, 6, 7, although the definition of inactive might be interpreted as healed (or healing). Similar to our previous study, interrater reliability for no ulcer versus inactive ulcer produced a negative kappa statistic, which suggests that the inclusion of a healed or inactive ulcer category is unlikely to be helpful in clinical trials with multiple raters. We also did not provide the raters with definitions of the categories or exemplar images, as we did not intend to test the reliability of a particular grading system. In addition, there are other clinical context factors that we did not include, which should be further explored (e.g., history of DUs and trauma). It could be argued that the high level of intrarater reliability is in part due to recall by the grader; however, each participant viewed a large number (n = 80) of unique images and the repeats were taken from the first 50, thereby reducing the likelihood of significant recall.

In conclusion, the addition of clinical context was not associated with a significant increase in either the overall intra‐ or interrater reliability of DU grading by rheumatologists. Agreement between the individual patients and rheumatologists was also poor. Further work to facilitate future SSc trials (incorporating international consensus) is needed, including (but not limited to) the development of DU grading systems (potentially encouraging clinicians to use clinical contextual information in particular to help distinguish no ulcer from DU, and including studies examining the impact of training), exploring the patient DU construct, and the investigation of (objective) measurement techniques.

## AUTHOR CONTRIBUTIONS

All authors were involved in drafting the article or revising it critically for important intellectual content, and all authors approved the final version to be submitted for publication. Dr. Hughes had full access to all of the data in the study and takes responsibility for the integrity of the data and the accuracy of the data analysis.

**Study conception and design.** Hughes, Roberts, Tracey, Dinsdale, Murray, Herrick.

**Acquisition of data.** Hughes, Tracey, Herrick.

**Analysis and interpretation of data.** Hughes, Roberts, Dinsdale, Murray, Herrick.

## Supporting information

Supplementary Table 1. Patient and digital lesion characteristics
