# Supplementary material for: Does the Clinical Context Improve the Reliability of Rheumatologists Grading Digital Ulcers in Systemic Sclerosis?
Source: Arthritis Care Res (Hoboken). 2016 Jul 28;68(9):1340–5. doi: 10.1002/acr.22833 (PMC5006886; doi:10.1002/acr.22833)
Supplement: Supplementary file 1 — Supplementary Table 1. Patient and digital lesion characteristics [file ACR-68-1340-s001.doc]

| **Supplementary Table 1. Patient and digital lesion characteristics** | | |
| --- | --- | --- |
| **Patient characteristics** (n = 36) | |  |
| Sex: female (number, %) | | 28 (80%) |
| Age in years, mean (SD) | | 56.4 (14.1) |
| Disease subtype (limited) | | 19 (54%) |
| RP duration in years, mean (SD) * | | 18.2 (14.6) |
| Disease duration in years, mean (SD) * | | 12.4 (10.8) |
| History of intravenous vasodilator therapy, number (%) | | 18 (51%) |
| History of debridement, number (%) | | 14 (40%) |
| History of amputation, number (%) | | 4 (11%) |
| **Digital lesion characteristics** (n = 80) | |  |
| Pain VAS (x/100), mean (SD) | | 36.3 (31.4) |
| Pain temporal (number) | Not applicable | 20 |
|  | Less | 15 |
|  | Same | 32 |
|  | Worse | 13 |
| Duration of the lesion (number) | <1 month | 12 |
|  | 1-3 months | 21 |
|  | 3-6 months | 7 |
|  | 6-9 months | 6 |
|  | 9-12 months | 3 |
|  | >12 months | 31 |
| Discharge: Patient reported, ‘yes’ * (number) | | 10 |
| Discharge: Clinician observed, ‘yes’ * (number) | | 6 |
